# Multiparametric mapping in the diagnosis and management of cardiac transplant rejection: a prospective, histologically-validated study

**DOI:** 10.1186/1532-429X-18-S1-O67

**Published:** 2016-01-27

**Authors:** Muhammad Imran, Louis Wang, Jane McCrohon, Cameron Holloway, James Otton, Chung-Yao Yu, Justyn Huang, Christian Stehning, Valentina Puntmann, Kirsten Moffat, Joanne Ross, Vassilis Vassiliou, Sanjay Prasad, Eugene Kotlyar, Anne Keogh, Christopher Hayward, Peter Macdonald, Andrew Jabbour

**Affiliations:** 1 Victor Chang Cardiac Research Institute, Sydney, NSW Australia; 2 Cardiology, St. Vincent's Hospital, Sydney, NSW Australia; 3 Kings College London, London, UK; 4 Philips research Hamburg, Hamburg, Germany; 5 Royal Brompton and Harefield, London, UK; 6 Heart Transplant unit, St. Vincent's Hospital, Sydney, NSW Australia; 7 Medical Imaging, St. Vincent's Hospital, Sydney, NSW Australia

## Background

Routine surveillance endomyocardial biopsies (EMBx) in heart transplant recipients are associated with serious complications. CMR-based tissue characterisation using T1 and T2 mapping sequences accurately and non-invasively diagnoses extracellular space expansion and interstitial oedema and theoretically, holds potential for the detection of acute cardiac rejection and chronic interstitial myocardial fibrosis associated with chronic rejection. This study aimed to determine the diagnostic role of novel T1- and T2-mapping sequences in heart transplant recipients.

## Methods

Patients underwent CMR within 24 hours of their surveillance or clinically-indicated EMBx starting from six weeks after transplantation. The native T1 maps were acquired using Modified Look-Locker Inversion recovery (MOLLI) sequence in a single mid-ventricular short axis slice (Philips Medical Systems, Best, Netherlands). T1 values were calculated by fitting an exponential curve to measured data from 8 different phases after manual offline motion correction. The T2 values were measured using a respiratory-navigated black-blood turbo spin echo sequence in a single mid-short-axis slice. The EMBx tissue was stained with Masson's trichrome stain to assess the degree of fibrosis and was graded for rejection degree according to International Society of Heart and Lung Transplantation (ISHLT) guidelines.

## Results

Of 84 scans, 42 biopsies were ISHLT grade 0 (group 0), 27 grade 1R (group 1), 2 grade 2R, 7 grade 3R or antibody mediated rejection and 6 clinically-diagnosed rejections (group 2). T1 values were significantly higher in group 2 (n = 15, 1033 (10.42); mean (SEM)) compared to group 0 (n = 27, 983 (7.18); p = 0.0001) and group 1 (n = 42, 995.2 (17. 06); p = 0.01). There was a strong correlation between T1 values measured exclusively in the interventricular septum vs circumferentially (R^2^ 0.886, p < 0.0001). Out of total 59 samples stained with Masson's trichrome stain, 18 had no visible fibrosis and 41 had quantifiable fibrosis; grade 1 (5-10%), grade 2 (10-30%) and grade 3 (>40%). The patients with higher grade interstitial fibrosis had higher T1 values (p < 0.05). The T2 values were also significantly higher in rejection group 2 (n = 13, 67.59 (1.95)) vs. group 1 (n = 26, 56.07 (1.266); p < 0.0001) and group 0 (n = 43, 53.60 (0.687); p < 0.0001).

Using 970 msec as a cut off on ROC analysis, T1 had sensitivity of 100% and specificity of 42% for the diagnosis of acute rejection using histology as the gold standard. Using a value of 60, T2 had sensitivity of 92% and specificity of 84%. The combination of T2 and T1 mapping resulted in a ROC curve area of 0.946 with 100% sensitivity, 85% specificity, a negative predictive value of 100% and a positive predictive value of 54% (low prevalence).

## Conclusions

Multiparametric mapping using CMR-based T1- and T2-mapping displays high sensitivity and specificity in the detection of cardiac allograft rejection and holds promise to reduce the dependency on invasive EMBx after cardiac transplantation.

**Figure 1:**
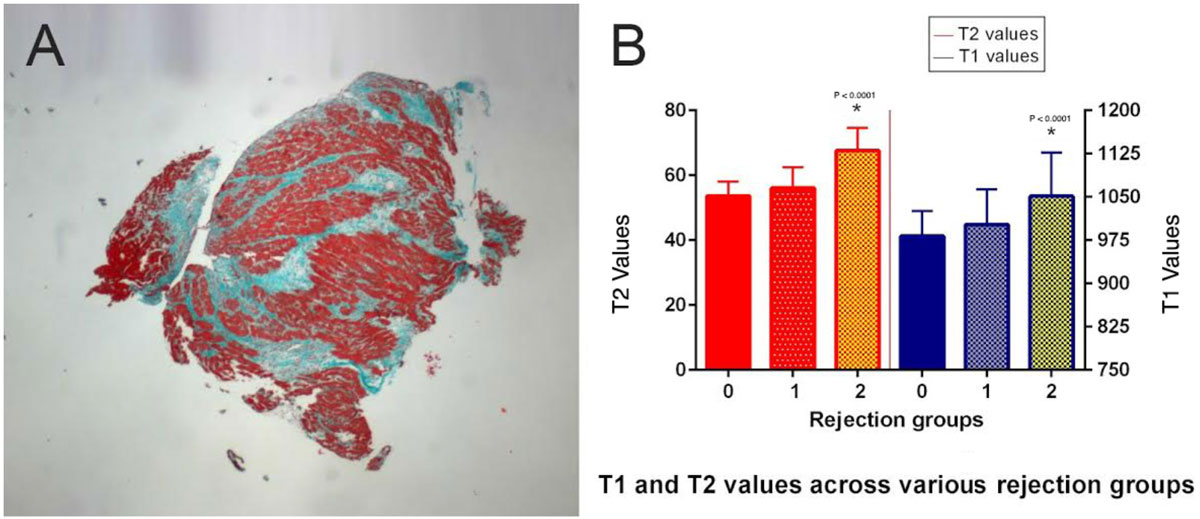
**A. EMBx tissue with Masson's Trichrome stain demonstrating interstitial myocardial fibrosis**. 1. B. Comparison of T1 and T2 values between various rejection groups. Group 0: ISHLT grade 0, Group 1: ISHLT grade 1R, Group 2: ISHLT grade 2R, 3R, antibody mediated rejection and clinically-diagnosed rejection.

**Figure 2:**
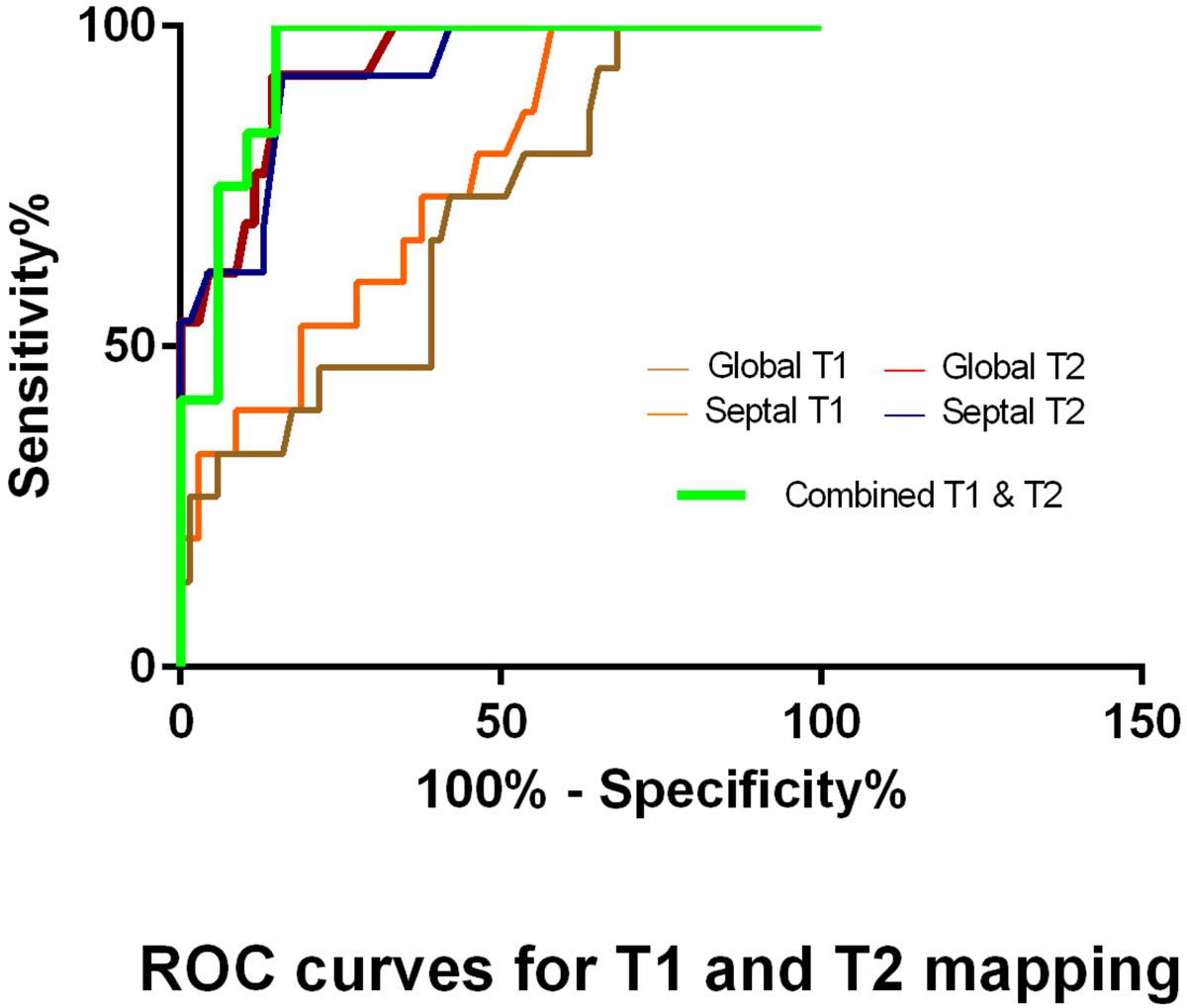
**ROC curves for T1 and T2 values measured along the interventricular septum (septal T1 & T2) as well as circumferentially at mid-ventricular short axis slice (Global T1 and T2) to diagnose rejection in heart transplant recipients**. The area under the curve for combined T1 and T2 values was 0.946 with sensitivity of 100% and specificity of 85%.

